# Targeting Nuclear Receptors for T_H_17-Mediated Inflammation: REV-ERBerations of Circadian Rhythm and Metabolism

**DOI:** 10.20900/immunometab20220006

**Published:** 2022-03-03

**Authors:** Sarah A. Mosure, Adrianna N. Wilson, Laura A. Solt

**Affiliations:** 1 Department of Immunology and Microbiology, The Scripps Research Institute, Jupiter, FL, 33458, USA; 2 Skaggs Graduate School of Chemical and Biological Sciences, The Scripps Research Institute, Jupiter, FL 33458, USA; 3 Department of Integrative Structural and Computational Biology, The Scripps Research Institute, Jupiter, FL 33458, USA; 4 Department of Molecular Medicine, The Scripps Research Institute, Jupiter, FL 33458, USA

**Keywords:** T_H_17 cell, nuclear receptor, RORγt, REV-ERB, T regulatory, inflammation, metabolism

## Abstract

Since their discovery, a significant amount of progress has been made understanding T helper 17 (T_H_17) cells’ roles in immune homeostasis and disease. Outside of classical cytokine signaling, environmental and cellular intrinsic factors, including metabolism, have proven to be critical for non-pathogenic vs pathogenic T_H_17 cell development, clearance of infections, and disease. The nuclear receptor RORγt has been identified as a key regulator of T_H_17-mediated inflammation. Nuclear receptors regulate a variety of physiological processes, ranging from reproduction to the circadian rhythm, immunity to metabolism. Outside of RORγt, the roles of other nuclear receptors in T_H_17-mediated immunity are not as well established. In this mini-review we describe recent studies that revealed a role for a different member of the nuclear receptor superfamily, REV-ERBα, in the regulation of T_H_17 cells and autoimmunity. We highlight similarities and differences between reports, potential roles beyond T_H_17-mediated cytokine regulation, unresolved questions in the field, as well as the translational potential of targeting REV-ERBα.

## INTRODUCTION

IL-17-producing CD4^+^ T helper cells, T_H_17 cells, play critical roles maintaining immune system homeostasis at mucosal barriers, responding to extracellular pathogens to clear infection [1]. However, T_H_17 cells have garnered considerable attention given that dysregulated T_H_17 responses can contribute to autoimmune disease and chronic inflammation, including multiple sclerosis (MS) and psoriasis [2,3]. A number of T_H_17 cell types have been identified ranging from non-pathogenic T_H_17 cells (T_H_17n) to pathogenic T_H_17 cells (T_H_17p). T_H_17n cells secrete IL-17 and IL-10 and work in an immune-modulating capacity in balance with forkhead box P3^+^ (Foxp3^+^) T regulatory (Treg) cells. T_H_17p cells secrete pro-inflammatory cytokines such as IL-17, interferon gamma (IFNγ), and granulocyte-macrophage colony-stimulating factor (GM-CSF) [4–6]. Their non-pathogenic or pathogenic potential can be initiated by cytokines in the milieu at the time of naïve CD4^+^ T cell activation [7]. Alternatively, cellular metabolic processes, including glycolysis and oxidative phosphorylation (OXPHOS), play significant roles in the developmental potential of T_H_17 cells [8,9]. Specifically, increased aerobic glycolysis is strongly associated with T_H_17 cell pathogenicity, and while OXPHOS is also elevated in T_H_17p, inhibition of glycolysis appears to be more effective in preferentially targeting T_H_17p vs T_H_17n cells [8,9]. Finally, a coordinated network of transcription factors, including basic leucine zipper transcriptional factor ATF-like (BATF) and interferon-regulatory factor 4 (IRF4) initiate chromatin remodeling enabling other transcription factors, like signal transducer and activator of transcription 3 (STAT3) and RAR-related orphan receptor gt (RORγt, NR1F3), to influence nuanced T_H_17 cell development and effector functions [10,11]. STAT3 orchestrates expression of RORγt, the lineage defining transcription factor for T_H_17 cells, collectively modulating effector function through induction of key RORγt/T_H_17 cell genes such *Il17a* and *Il23r* [10–12].

**Figure 1.**
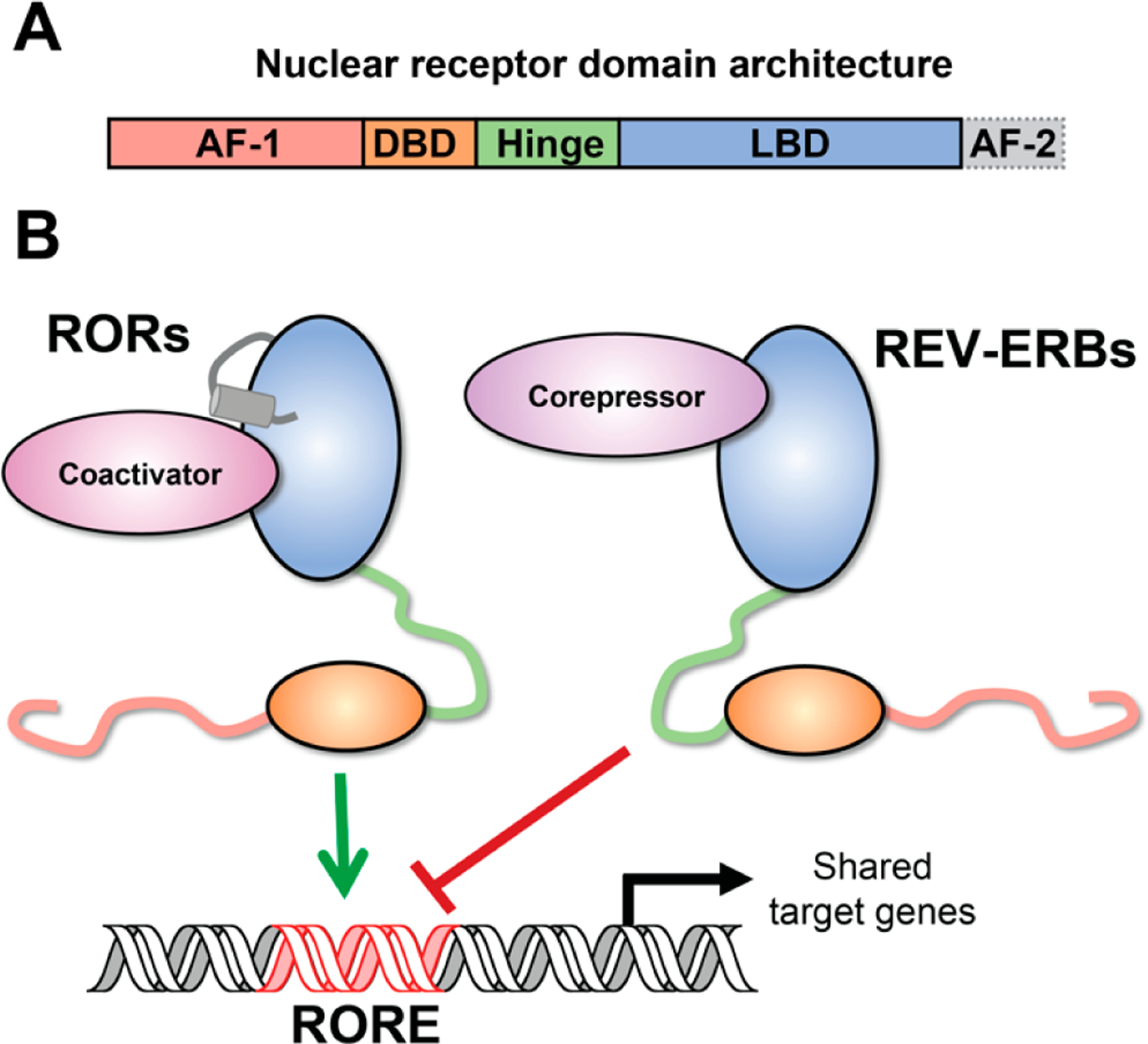
Nuclear receptor structure and function. (**A**) The conserved nuclear receptor domain architecture from N- to C-terminus includes the activation function-1 (AF-1) domain, which is thought to perform ligand-independent activities. The AF-1 is followed by the DNA binding domain (DBD), which recognizes, and binds target sites on DNA. The hinge region provides a flexible linker between the DBD and the LBD, which binds ligands and coregulator proteins. The LBD incudes the activation function-2 (AF-2) helix, which is critical for recruiting coactivator proteins. (**B**) Schematic depicting reciprocal regulation of shared target genes by RORs and REV-ERBs. RORs activate transcription at ROR response elements (ROREs) by recruiting coactivator proteins via their AF-2 helix. REV-ERBs compete for binding and represses transcription at these shared sites by recruiting corepressors (e.g., NCoR); since REV-ERBs lack the AF-2 helix, they cannot recruit coactivators or activate transcription.

RORγt is a member of the nuclear receptor (NR) superfamily of ligand regulated transcription factors. RORγt, however, is not the only NR associated with T_H_17 cell function. RORα (NR1F1), a close family member of RORγt, and REV-ERBα (NR1D1) have also been shown to be involved in the regulation of T_H_17 cell development and function [12–17]. REV-ERBa is encoded by the opposite DNA strand of the *ERBA* oncogene. Hence its name is derived from ‘reverse strand of *ERBA*’. NRs share a common core structure comprised of an amino terminus of variable length, a highly conserved central DNA binding domain (DBD), a lesser conserved ligand binding domain (LBD), and a flexible hinge region located between the DBD and LBD regions which often contains the nuclear localization sequence [18] (Figure 1A). Despite the high degree of sequence similarity of NRs, largely in their DBDs, they are functionally diverse through their differential recruitment of coregulators and subsequent chromatin remodelers. NRs interact with coregulators through both ligand-independent and dependent mechanisms. Specific coregulator interaction enables NR-target gene transcription. Binding of endogenous RORγt agonist ligands such as oxysterols and other cholesterol metabolites can increase recruitment of coactivator steroid receptor coactivator-1 (SRC-1) increasing chromatin accessibility at RORγt DNA recognition elements [19,20]. SRC-3 has been shown to be required for RORγt-mediated T_H_17 cell pathogenicity [21]. Due to their pro-inflammatory role in several autoimmune and chronic inflammatory diseases, T_H_17 cells and RORγt have been a pharmacological target for over a decade. RORγt’s translational potential has been exploited by numerous pharmaceutical companies. To date, approximately 20 candidate compounds have entered clinical trials [22]. Unfortunately, most of the candidates were either discontinued or suspended for further development due to safety concerns or lack of clinical efficacy [22]. Therefore, there is a need to understand these concerns and identify other potential therapeutic targets for the treatment of T_H_17-mediated inflammatory diseases.

## REGULATION OF T_H_17 CELLS BY THE NUCLEAR RECEPTORs REV-ERBα and REV-ERBβ

The REV-ERBs, REV-ERBα and REV-ERBβ (NR1D2), are two members of the NR superfamily and highly conserved proteins. Unlike most NRs, the REV-ERBs lack the conserved C-terminal helix necessary to recruit coactivator proteins and therefore interact exclusively with corepressors, including Nuclear Receptor Corepressor (NCoR). As a result, the REV-ERBs exclusively repress transcription. The REV-ERBs share a DNA response element, termed a RORE (ROR-response element), with the ROR NRs. Whereas the RORs activate, the REV-ERBs repress target gene transcription at these sites (Figure 1B). This opposing activity ensures temporal control of target gene expression in tissues where the REV-ERBs and RORs are co-expressed, including brain, liver, adipose tissue, and skeletal muscle [23]. This coordinated regulation of shared target genes by the RORs and REV-ERBs contributes to the circadian rhythm in mammals. The circadian rhythm is comprised of feedback loops of proteins that make up the molecular clock. Heterodimers of the transcription factors brain and muscle ARNT-like 1 (BMAL1) and circadian locomotor output cycles protein kaput (CLOCK), known as the positive limb of the circadian clock, induce the expression of the negative limb, cryptochrome (*CRY1* and *CRY2*) and period (*PER1*, *PER2*, and *PER3*) circadian clock genes. As CRY and PER reach critical levels in the cell, they repress the expression of BMAL1/CLOCK heterodimers, thus downregulating their transcriptional activity. The RORs and REV-ERBs form an essential accessory loop resulting in further positive and negative regulation of gene transcription, respectively. Importantly, they co-regulate genes in the core circadian clock, including BMAL1. The expression of these proteins oscillates over the course of a 24 h period and regulate the expression of cell type-specific target genes to produce rhythmic expression [24]. These circadian processes have been well defined in several cell types including liver, skeletal muscle, and adipose tissue. While it remains unclear whether T cells undergo circadian regulation, some evidence suggests the REV-ERBs and RORs together with Nuclear Factor Interleukin 3 Regulated (NFIL3) exert circadian regulation of T_H_17 cells [17,25,26].

Recent evidence from our lab and others have shown that the REV-ERBs and RORs are also co-expressed in T_H_17 cells [14,16,17]. REV-ERBα in particular exhibits T_H_17 cell-specific expression relative to the other T helper subtypes. In line with REV-ERBα’s role as a repressor, REV-ERBα-deficient (*Nr1d1*^*−/−*^) T_H_17 cells exhibit increased expression of core RORγt/T_H_17 cell genes, including *Il17a*, *Il17f*, and *Il23r,* when assessed by RNA-sequencing [14]. Reciprocally, overexpression of REV-ERBα results in repression of these core target genes [14,16]. Mechanistically, ChIP-sequencing and ChIP-qPCR data show REV-ERBα directly competes with RORγt for binding at shared target sites within the regulatory elements of core T_H_17 cell genes, including *Il17a* and *Il23r* [14,16]. Using in vivo models of T_H_17 cell-mediated autoimmunity, including experimental autoimmune encephalomyelitis (EAE), a mouse model of multiple sclerosis, global REV-ERBα deficiency exacerbates disease severity by increasing CD4^+^ T cell number and pro-inflammatory cytokine expression in the central nervous system (CNS). It is important to note that increased disease scores in REV-ERBa deficient mice could be a consequence of general disruption of the circadian system, which is associated with higher inflammation levels [27]. Similar results to the EAE model were observed in colons of *Rag1*^*−/−*^ mice, which are devoid of T and B cells, receiving *Nr1d1*^*−/−*^ T cells in an adoptive transfer model of colitis [14]. In this model intraperitoneally delivered T cells are activated by gut microbes to elicit inflammation that models human colitis. In contrast, a separate study demonstrated T-cell specific loss of both REV-ERBα and REV-ERBβ leads to decreased T_H_17 cells and disease score in mice with EAE [16]. This data is consistent with work published several years earlier demonstrating a link between the circadian clock and T_H_17 cell development [17]. Intriguingly, induction of REV-ERBα expression in REV-ERBα-deficient T_H_17 cells delays EAE onset and limits disease progression [16]. Overall, these findings indicate REV-ERBα competes with RORs for binding at regulatory elements within core T_H_17 cell genes and differential expression of REV-ERBα can restrain T_H_17 cell pathogenicity.

Given the REV-ERBs are members of the NR family of ligand-regulated transcription factors, they are amenable to regulation by small molecule ligands [23,28–30]. Indeed, several synthetic ligands have been reported to modulate REV-ERB activity. SR9009 and SR12418 enhance REV-ERB-dependent target gene repression and have sufficient in vivo exposure to interrogate ligand-dependent activity in vivo [14,16]. Despite the conflicting genetic data, consistent with their expected role in enhancing REV-ERB-dependent repression, REV-ERB ligands limit disease progression in both chronic and relapsing-remitting models of EAE by inhibiting pro-inflammatory T_H_17 cell development, migration, and effector function in the CNS [14,16,17]. Although some studies have suggested REV-ERB-mediated target gene repression may be saturated due to their basal repressive activity or the presence of their endogenous ligand, these data using synthetic ligands indicate REV-ERB activity is not saturated in T_H_17 cells. Furthermore, mechanistic studies showed these ligands operate by enhancing corepressor recruitment at target sites including the *Il17a* locus [16]. Although these studies provide compelling evidence that REV-ERB ligands modulate T_H_17 cell activity, recent work has suggested that SR9009 has REV-ERB-independent effects on cellular metabolism. However, direct experimental evidence supports the conclusion that SR9009-dependent activity in T_H_17 cells is specific to REV-ERB modulation since ligand-dependent repression of IL-17A is lost in REV-ERBα/β double knockout (DKO) T_H_17 cells [16]. Our group has also successfully recapitulated these experiments (unpublished data); however, we acknowledge this is a single target gene and whether more global transcriptional changes are specific to ligand-mediated REV-ERB repression will need to be explored in greater detail. Going forward, it will be important to investigate whether newer generations of REV-ERB ligands with greater potency and specificity ameliorate disease in in vivo models of T_H_17-mediated autoimmunity [28,31]. Furthermore, it will be critical to include REV-ERBα/β DKO controls for all future experiments involving REV-ERB ligands.

Although published data defining a role for REV-ERBα in T_H_17 cells has been limited to mouse models, recent findings also support a role for REV-ERBα in human T_H_17 cells. REV-ERBα was identified as a T cell-specific MS susceptibility gene in a GWAS study from the MS Consortium, and REV-ERBα was among the genes differentially expressed in T_H_17p cells derived from human patients [32,33]. Collectively, these findings indicate REV-ERBα is a core regulator of T_H_17 cell-mediated autoimmunity in both mice and humans and therefore may be a viable target for small molecule therapeutics. This is important given that current therapeutics for autoimmune and chronic inflammatory diseases such as corticosteroids and aminosalicylates have negative side effects or variable efficacy [34–36]. Furthermore, results from the RORγt modulator clinical trials have been disappointing [22]. Thus, there is a need for more treatment options and targeting REV-ERBα could be a new opportunity worth pursuing. However, since REV-ERBs are globally expressed and important for maintaining the circadian rhythm, it may be necessary to simultaneously pursue T_H_17 cell-specific targeted delivery of REV-ERB ligands (i.e., through antibody-drug conjugates) to avoid deleterious off-target effects [37]. It may also be important to consider the timing of therapeutic administration given the possibility that REV-ERB expression in T_H_17 cells could undergo circadian fluctuations.

Genetic and pharmacological studies indicate a role for REV-ERBα as a repressor of T_H_17 cell pathogenicity; however, a role for its closely related sister protein REV-ERBβ remains unclear. Intriguingly, while REV-ERBα and REV-ERBβ expression and activity has been shown to be redundant in most tissues, T_H_17 cells appear to be unique in that the two REV-ERBs exhibit differential expression and activity. Although overexpression of REV-ERBβ largely phenocopies overexpression of REV-ERBα, it remains unclear how REV-ERBβ deficiency affects T_H_17 cell activity. Current evidence includes T_H_17 cells deficient in both REV-ERBs (REV-ERBα/β deficient), an EAE model using the T cell-specific REV-ERBα/β DKO mice, and a mouse model of circadian disruption assessing intestinal T_H_17 cell frequencies [16,17]. REV-ERBα/β DKO T_H_17 cells had the opposite phenotype of REV-ERBα single deficient cells and REV-ERBα deficient mice such that disease was ameliorated by loss of both REV-ERBs [16]. This contradicts the repressive effect of REV-ERBα and REV-ERBβ overexpression, as well as the repressive effect of REV-ERB ligands, which are expected to activate both REV-ERBs. One confounding variable that could underlie this discrepancy is the repression of REV-ERBb by REV-ERBa such that REV-ERBa knockout results in higher REV-ERBb expression. However, REV-ERBa itself, as well as REV-ERBβ, have been demonstrated to negatively regulate REV-ERBa [38,39]. Given this information, it is possible that loss of REV-ERBb leads to increased REV-ERBa expression thereby presenting an alternative hypothesis for the data currently at hand. Thus, more comprehensive experiments are needed to define the unique role for REV-ERBβ in T_H_17cells.

Outside of the immune system, the REV-ERBs and RORs participate in regulating the circadian clock such that many of their tissue-specific target genes undergo rhythmic expression [23]. Much like the genetic data surrounding REV-ERBα in T_H_17 cells, there is conflicting evidence as to whether adaptive immune responses, including T cell gene expression and effector responses, are affected by circadian rhythms [25,26,40]. Previous work showed that T_H_17 cells in particular are strongly influenced by the circadian clock. This seems logical given the lineage-defining transcription factor in T_H_17 cells, RORγt, is an isoform of a core circadian protein (RORγ). In hepatocytes, RORγ is thought to be the dominant circadian factor driving rhythmic gene expression [41–43]. The study exploring a role for REV-ERBα in circadian T_H_17 cell activity found loss of REV-ERBα inhibits T_H_17 cell differentiation [17]. REV-ERBα worked in concert with another circadian protein, NFIL3 (also known as E4BP4), to control RORγt expression and thus, T_H_17 cell development. The discrepancy between this finding and our published data could be due to differences in microbiota between the mouse facilities or the genetic background of the REV-ERBα-deficient mice, which were engineered differently. Regardless, both point to a critical role for REV-ERBα in regulating T_H_17 cell activity and a role for the circadian clock in adaptive immunity.

A large body of evidence indicates that metabolic genes are regulated by the circadian clock [44,45]. Indeed, REV-ERBα has been shown to orchestrate circadian control of metabolic genes in liver, adipose tissue, and skeletal muscle [23]. In the liver, changes in glucose availability affect the synthesis of the natural REV-ERB ligand, heme. Heme subsequently enhances REV-ERBα repression of metabolic genes, forming a negative feedback loop. Intriguingly, changes in metabolism are also a hallmark feature of T_H_17p vs T_H_17n cells. T_H_17p cells exhibit an overall increase in metabolism, particularly glycolysis, as well as changes in fatty acid composition [9,46–48]. Changes in fatty acid composition have been shown to influence RORγt activity through modulation of the RORγt ligand pool, which subsequently increases pathogenic gene expression [48]. Whether REV-ERBα is similarly regulated by changes in metabolism in T_H_17 cells (i.e., through modulation of heme or lipid synthesis) requires further investigation. At the same time, REV-ERBα activity has been shown to enhance oxidative phosphorylation and mitochondrial biogenesis in muscle tissue [49]. To our knowledge, the effect of REV-ERBα deficiency or ectopic overexpression on T_H_17 cell metabolism remains to be explored. For such studies, it will be important to compare effects in T_H_17n vs T_H_17p cells in vitro, as well as in vivo*-*derived cells due to the significant metabolic differences reported for in vitro vs in vivo T cells [50,51]. It will also be important to include REV-ERB ligands in these studies to determine whether effects can be ligand-regulated. These experiments exploring whether REV-ERBα contributes to the regulation of T_H_17 cell metabolism could uncover exciting new avenues in our understanding of T_H_17 cell activity.

## PERSPECTIVE

T_H_17 cells are a central driver of several autoimmune and chronic inflammatory diseases, many of which are in need of safer and more effective treatment options. Thus, a better understanding of the factors that globally regulate the T_H_17 cell phenotype is paramount to developing new therapeutics. Recent evidence has shown pathogenic T_H_17 cells undergo metabolic remodeling to meet their increased demand for energy and biomolecular building blocks. This observation has led to the idea that T_H_17p cells can be specifically targeted with therapeutics aimed to inhibit these upregulated processes. At the same time, work from our lab and others have identified REV-ERBα as a critical regulator of T_H_17 cell pathogenicity. This experimental evidence from mouse models is supported by genetic evidence from human patients which also found REV-ERBα expression is associated with disease susceptibility. Excitingly, REV-ERBα is amenable to ligand regulation and small molecule ligands that enhance REV-ERBα activity ameliorate disease in several models of autoimmunity. Given that REV-ERBα has been shown to regulate metabolic processes in most tissues in which it is expressed (i.e., liver, adipose tissue, and skeletal muscle), it is not unlikely that REV-ERBα may also regulate T_H_17 cell metabolic processes. Thus, we propose that efforts to better understand and target REV-ERBα-mediated regulation of T_H_17 cell activity and metabolism would complement current campaigns to directly target T_H_17p cell metabolism. However, given the conflicting genetic evidence regarding the role of REV-ERBα in T_H_17 cell activity, further studies are warranted to resolve these contradictions. Investigations into REV-ERBα regulation by its natural ligand, heme, in T_H_17 cells could also uncover new pathways linking REV-ERBα activity and metabolism. Overall, the current evidence suggests REV-ERBα is a compelling regulatory factor in T_H_17 cells, and deeper exploration of REV-ERBα activity could offer a better understanding of T_H_17 cell biology as well as new therapeutic opportunities.

## Figures and Tables

**Figure 2.**
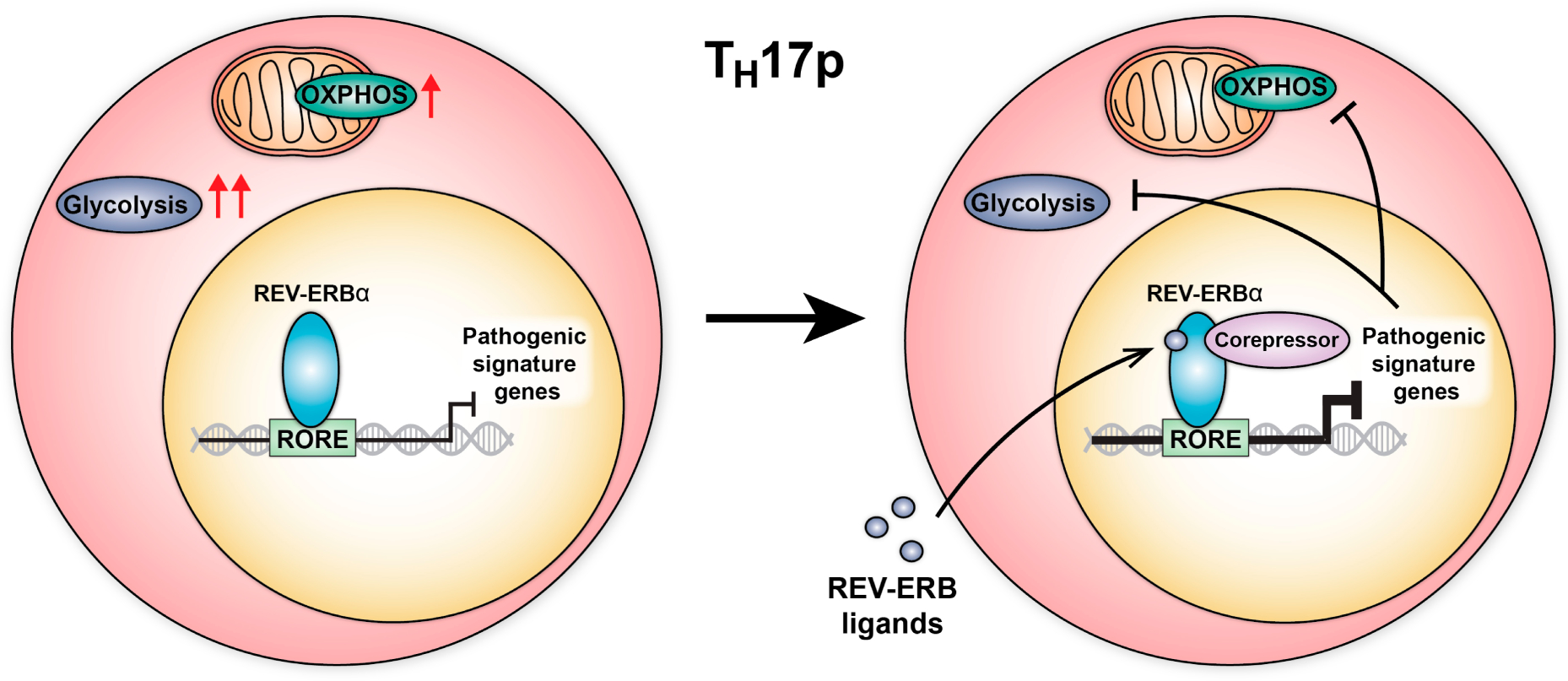
Proposed mechanism for ligand-dependent REV-ERBα activity in inhibiting metabolism in T_H_17p cells.

## ABBREVIATIONS

T_H_17: T helper 17
T_H_17n: non-pathogenic T_H_17
T_H_17p: pathogenic T_H_17
NR: nuclear receptors
DBD: DNA-binding domain
LBD: ligand-binding domain
MS: multiple sclerosis
EAE: experimental autoimmune encephalitis
Treg: T regulatory cell
OXPHOS: oxidative phosphorylation
ROR: RAR-related orphan receptor
STAT: signal transducer and activator of transcription
REV-ERBα: reverse strand of ERBA
Foxp3: forkhead box p3
NFIL3: Nuclear Factor Interleukin 3 Regulated
BMAL1: brain and muscle ARNT-like 1
CLOCK: circadian locomotor output cycles protein kaput
PER1: Period 1
CRY1: Cryptochrome 1
NCoR: Nuclear Receptor Corepressor
